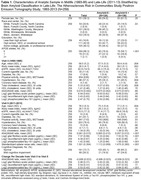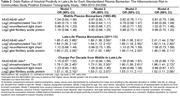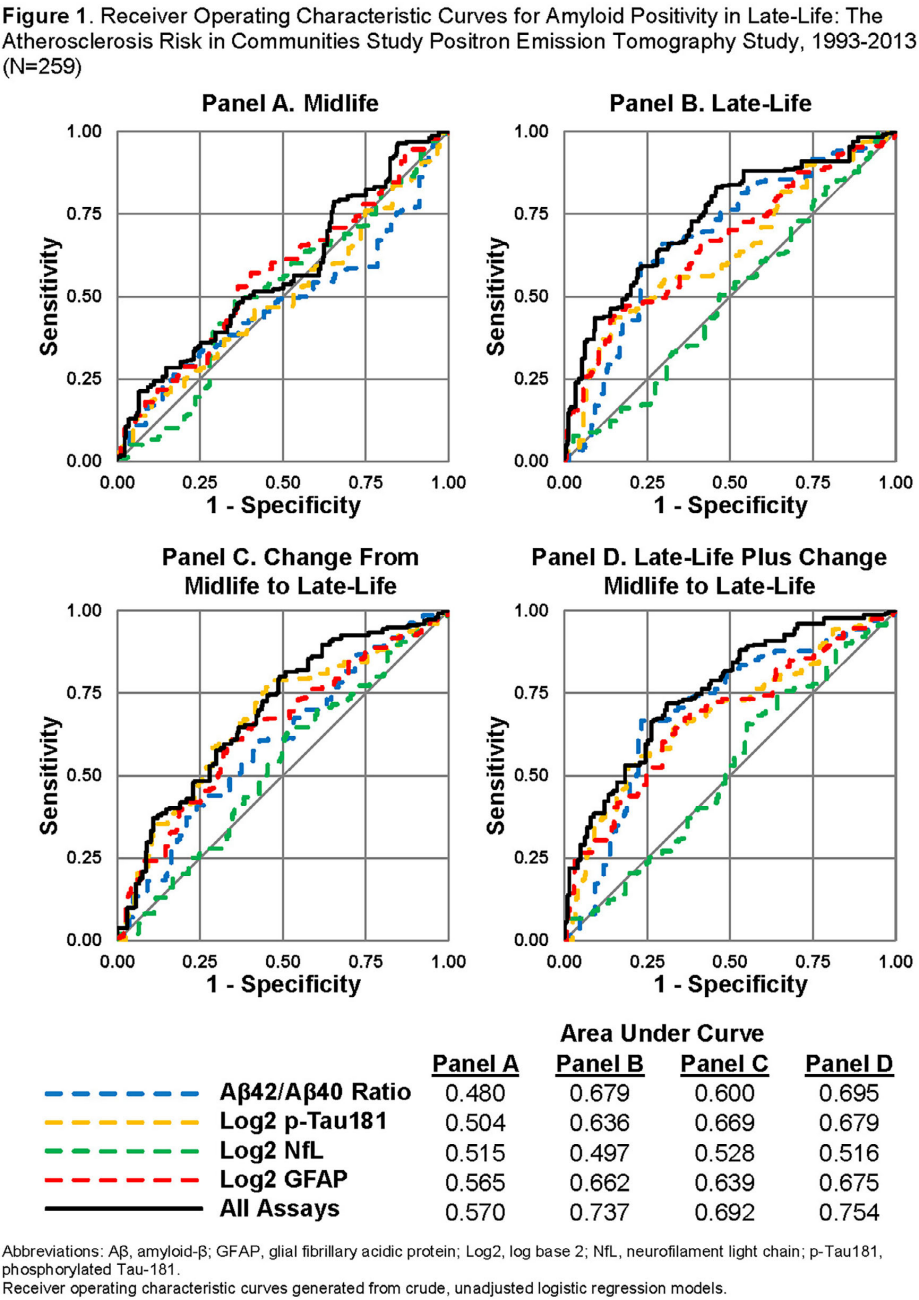# Mid‐ to late‐life changes in blood‐based biomarkers of Alzheimer’s disease pathology and neurodegeneration and associations with brain amyloid deposition: The ARIC‐PET Study

**DOI:** 10.1002/alz.091307

**Published:** 2025-01-09

**Authors:** James Russell Pike, Yifei Lu, Jinyu Chen, Keenan A. Walker, Kevin J. Sullivan, Michael E. Griswold, Bharat Thyagarajan, Michelle M. Mielke, Pamela L. Lutsey, Dean F. Wong, David S. Knopman, A. Richey Sharrett, Josef Coresh, Thomas H. Mosley, Rebecca F. Gottesman, Priya Palta

**Affiliations:** ^1^ New York University, New York, NY USA; ^2^ University of North Carolina Chapel Hill, Chapel Hill, NC USA; ^3^ Laboratory of Behavioral Neuroscience, National Institute on Aging, Intramural Research Program, Baltimore, MD USA; ^4^ University of Mississippi Medical Center, The MIND Center, Jackson, MS USA; ^5^ University of Minnesota, Minneapolis, MN USA; ^6^ Wake Forest University School of Medicine, Winston‐Salem, NC USA; ^7^ University of Minnesota School of Public Health, Minneapolis, MN USA; ^8^ Washington University in St. Louis School of Medicine, St. Louis, MO USA; ^9^ Mayo Clinic, Rochester, MN USA; ^10^ Johns Hopkins University Bloomberg School of Public Health, Baltimore, MD USA; ^11^ University of Mississippi Medical Center, Jackson, MS USA; ^12^ National Institute of Neurological Disorders & Stroke Intramural Research Program, National Institute of Health, Bethesda, MD USA

## Abstract

**Background:**

Blood‐based biomarkers show promise as a noninvasive, inexpensive method for measuring Alzheimer’s disease pathology throughout the lifecourse. However, the predictive and classification accuracy of these biomarkers at different stages of the lifecourse and among diverse, community‐dwelling populations requires further investigation.

**Method:**

Between 2014 and 2015, 329 dementia‐free participants from the Atherosclerosis Risk in Communities Study underwent brain magnetic resonance imaging (MRI) and florbetapir positron emission tomography (PET). Amyloid positivity was defined as a global cortex standardized uptake value ratio greater than 1.2. Stored plasma samples collected in midlife (1993‐95, mean age 58.5 years) and late‐life (2011‐13, mean age 76.2 years) from a subsample of 259 participants were assayed in 2022 using Quanterix SiMoA. The assay quantified amyloid‐β (Aβ)42/40, phosphorylated tau at threonine 181 (p‐Tau181), neurofilament light (NfL), and glial fibrillary acidic protein (GFAP). Unadjusted and covariate‐adjusted logistic regression models estimated the association between plasma biomarkers from midlife and late‐life and amyloid positivity in late‐life. Unadjusted receiver operating characteristic curves documented the classification accuracy of the plasma biomarkers separately and collectively.

**Result:**

The sample (Table 1) included 151 women (58.1%), 105 Black participants (40.4%), and 136 (52.3%) participants with amyloid positivity. In models adjusted for demographics, lifestyle factors, cardiovascular factors, and the presence of APOE ε4 alleles, assays measured in midlife were not associated with amyloid positivity in late‐life (Table 2). However, late‐life measurements of Aβ42/40, p‐Tau181, and GFAP and change per decade from midlife to late‐life in p‐Tau181 and GFAP were associated with greater odds of amyloid positivity. The greatest classification accuracy (Figure 1) was achieved by using all assays measured in midlife and late‐life (AUC = 0.754), although the accuracy when using only late‐life measures was comparable (AUC = 0.737).

**Conclusion:**

Late‐life plasma biomarkers of Alzheimer's disease neuropathology and astrogliosis were associated with late‐life amyloid positivity, but midlife plasma biomarkers obtained 16 to 20 years earlier did not exhibit a statistically significant association. Additional longitudinal research is needed to determine whether changes in plasma biomarkers measured before an individual is classified as amyloid positive can be used to identify individuals at risk of developing Alzheimer's disease.